# Follicular dendritic cell sarcoma of the right tonsil: A case report and literature review

**DOI:** 10.3892/ol.2014.2726

**Published:** 2014-11-21

**Authors:** ZHONG-JIE LU, JI LI, SHUI-HONG ZHOU, LI-BO DAI, SEN-XIANG YAN, TING-TING WU, YANG-YANG BAO

**Affiliations:** 1 Department of Radiotherapy, The First Affiliated Hospital, College of Medicine, Zhejiang University, Hangzhou, Zhejiang 310003, P.R. China; 2 Department of Otolaryngology, The First Affiliated Hospital, College of Medicine, Zhejiang University, Hangzhou, Zhejiang 310003, P.R. China

**Keywords:** follicular dendritic cell sarcoma, tonsil, treatment, prognosis, size of tumor

## Abstract

The current study presents a case of extranodal follicular dendritic cell sarcoma (FDCS) of the tonsil and reviews the relevant literature. In the present case, a 59-year-old male presented with a globus sensation in the right pharynx for 6 weeks. On clinical examination, a painless non-ulcerated enlarged right tonsil was identified; the tonsil was covered with a normal mucus membrane. A right tonsillectomy was performed under general anesthesia. The final pathological diagnosis was follicular dendritic cell sarcoma of the right tonsil. Postoperatively, the patient received radiotherapy. The patient remains alive without disease recurrence or metastasis 44 months after tonsillectomy. To the best of our knowledge, only 42 cases of FDCS of the tonsil have been reported to date. Of the 42 cases, 41 patients underwent surgery and one patient refused treatment. A total of 23 (54.7%) received surgery alone. Adjuvant treatment was administered for 18 patients (42.9%). Six patients (14.3%) experienced local recurrences and two patients (4.8%) succumbed to the disease 24 months after treatment. The three-, five-, and eight-year overall survival rates for the entire group were 86.5, 77.8 and 77.8%, respectively. Furthermore, a tumor diameter of ≥4 cm was prognostic upon univariate analysis (χ^2^=4.634; P=0.031; excluding incomplete data). Tonsillar FDCS is rare and is associated with high rates of recurrence and metastasis, therefore, adjuvant treatment should be prescribed.

## Introduction

Follicular dendritic cell sarcoma (FDCS) is a rare tumor, of which ~200 cases (116 in the head and neck) have been reported in the English literature (1,2) since Monda *et al* (3) first described the condition in 1986. Extranodal FDCS of the head and neck occurs principally in the tonsils. To date, only cases in the English literature have been reviewed. In the present study, the Chinese literature regarding tonsillar FDCS is also reviewed.

The optimal therapeutic modality remains controversial as tonsillar FDCS is extremely rare. Although a wide surgical approach may be curative in certain patients, recurrences have been reported (1,2). The efficacy of adjuvant therapy (chemotherapy or radiation) remains unclear (1,2). De Pas *et al* (4) reported that chemoradiotherapy was ineffective when used to improve disease-free survival following radical tumor excision in 143 DCS cases with varying anatomical locations, which had been described in the English literature (4). Hu *et al* (2) reviewed 52 cases of FDCS in the pharyngeal region and found that postoperative adjuvant therapy increased disease-free patient survival compared with surgery alone (2). The identification of an optimal treatment modality requires further investigation using larger numbers of FDCS cases.

The current study presents a case of extranodal FDCS in the tonsil and reviews the relevant English and Chinese literature. Written informed consent for the publication of this study was obtained from the patient.

## Case report

**Figure 1:**
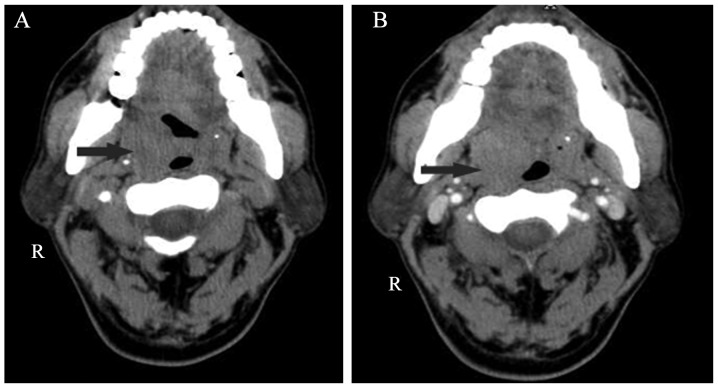
Computed tomography scans revealing a homogenously enlarged 4.6×2.5×2.5-cm right tonsil, which was well-circumscribed. (A) The initial enhancement value was 53 HU, and (B) slight continuing heterogeneous enhancement was evident following injection of contrast medium. R, right.

**Figure 2:**
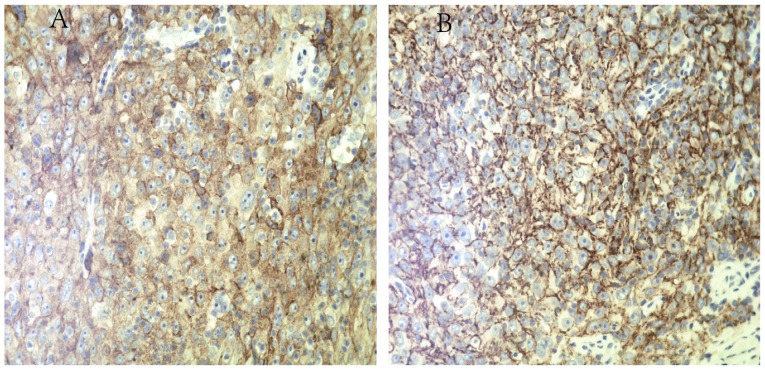
Pathological tests results showing a lesion containing large spindle-shaped heterogeneous cells forming solid or nested patterns, infiltrating the lymphoid stroma. Immunohistochemically, the cells were positive for (A) cluster of differentiation (CD)21 and (B) CD23.

In March 2011, a 59-year-old male presented to the Department of Otolaryngology, The First Affiliated Hospital, College of Medicine, Zhejiang University (Hangzhou, China) with a globus sensation in the right pharynx that had persisted for six weeks. There was no history of fever, dysphagia, odynophagia, dyspnea, otalgia, hoarseness or trismus. The patient had a smoking history of >20 years (10 cigarettes per day) and had consumed alcohol (500 ml per day) for >30 years, however, the remaining medical history was uneventful. Upon clinical examination, a painless, non-ulcerated, enlarged right tonsil was identified, which was covered with a normal mucosal membrane. The nasopharynx, tongue, hypopharynx, larynx and cervical lymph nodes were normal. The initial clinical diagnosis was of right tonsillar lymphoma. Pharyngeal computed tomography (CT) revealed a homogenously enlarged 4.6×2.5×2.5-cm right tonsil, which was well-circumscribed. The initial enhancement value was 53 HU, and slight continuing heterogeneous enhancement was evident following the injection of contrast medium. The cervical lymph node was not enlarged (Fig. 1). The CT observations indicated that the tumor could be a lymphoma. Ultrasonography of the abdomen and neck, and chest X-rays were unremarkable. A right tonsillectomy was performed under general anesthesia. Histological examination of frozen sections revealed the existence of a poorly-differentiated malignant tumor. Postoperative pathological tests showed that the lesion contained large spindle-shaped heterogeneous cells forming solid or nested patterns, infiltrating the lymphoid stroma. Immunohistochemically, the cells were positive for cluster of differentiation (CD)21 and CD23, and negative for CD3, CD10, CD20, CD30, CD35, anaplastic lymphoma kinase, and B-cell lymphoma 6. The Ki-67 index was 20% (Fig. 2). Thus, FDCS of the right tonsil was diagnosed.

**Figure 3:**
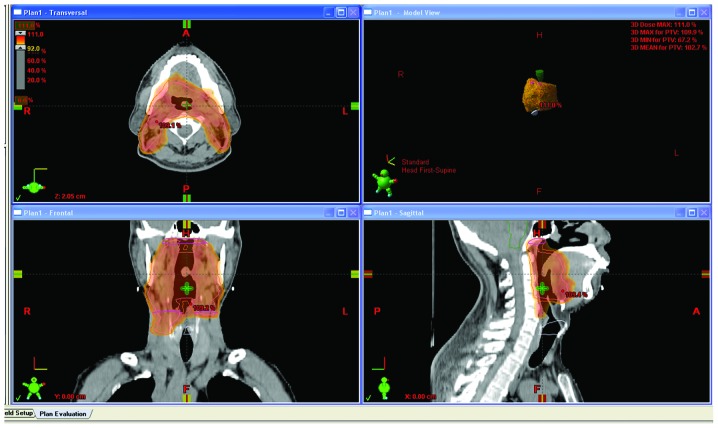
Postoperatively, the patient received radiotherapy (6,000 cGy in 200-cGy fractions delivered over 30 days) to the oropharyngeal and corresponding neck lymphatic regions.

Postoperatively, the patient received radiotherapy (6,000 cGy in 200-cGy fractions over 30 days) to the oropharyngeal and neck lymphatic regions (Fig. 3). The patient remains alive without disease recurrence or metastasis 44 months after undergoing the tonsillectomy.

## Discussion

Extranodal FDCS is rare. The etiology and the optimal treatment for extranodal FDCS remain unclear. In the present study, the English literature was reviewed using MEDLINE to conduct a PubMed/Web of Science search using the terms ‘follicular dendritic cell tumor’ or ‘follicular dendritic cell sarcoma’ combined with ‘extranodal’ or ‘head and neck/tonsil/oropharynx/pharynx/pharyngeal region’ (http://www.ncbi.nlm.nih.gov/pubmed). Articles published in the Chinese literature were found by searching the Wanfang (www.wanfangdata.com.cn), China National Knowledge Infrastructure (http://www.cnki.net/) and Weipu (http://10.15.61.77/index.asp) databases for studies published between 1986 and 2013. A total of 42 cases (including the present case) of FDCS were reported involving the tonsils; 29 cases were reported in the English literature (Table I) (2–27) and 13 cases were reported in the Chinese literature (Table II) (28–38). In the English literature, the study by Chan *et al* (27) was the first to describe tonsillar FDCS in 1994 (27). In the Chinese literature, the first case of FDCS of the tonsil was reported by Shi *et al* in 2004 (28). A decade ago, the disease entity was not well-documented and few immunohistochemical studies had been conducted (13).

Of the 42 patients reported, 22 were female and 19 were male; in one case, the gender of the patient was not reported. Of all the cases reported in the English literature, 15 patients were female and 14 were male. In the Chinese literature seven patients were female and five were male. The overall male to female ratio was ~1.16:1 (English literature, 1.07:1; Chinese literature, 1.4:1). Similar to the results reported by Duan *et al* (9), the overall mean patient age was 48 years (range, 18–80 years) at initial presentation (English literature: mean, 50.0 years and range, 18–76 years; Chinese literature: mean, 43.5 years and range, 19–80 years); the age of one patient was not reported,. A total of 14 tumors were located in the left tonsil, while 20 were located in the right tonsil; the affected tonsil was not reported in eight cases. The mean tumor length was 3.1 cm (range, 0.8–6 cm), however, no data were available for 14 patients.

A total of 41 patients underwent surgery and one refused treatment. Of the 42 patients, 23 (54.7%) received surgery alone. Adjuvant treatment was administered for 18 patients (42.9%). This included postoperative radiotherapy for 17 patients (40.5%), pre-operative radiotherapy for one patient (2.4%), postoperative chemotherapy for four patients (9.5%) and postoperative chemotherapy with radiotherapy for one patient (2.4%). One patient received doxorubicin and ifosfamide (8), and one patient was administered a cyclophosphamide, Adriamycin, vincristine and prednisone regimen (24,25). Details regarding the chemotherapy treatment were not provided in any studies from the Chinese literature. A total of five patients (11.9%) underwent neck dissection.

Follow-up data were available for 36 patients (85.7%), and the mean duration of follow-up was 36.6 months (range, 4 months to 15 years). A total of six patients (14.3%) experienced local recurrence. At the final follow-up after treatment, 25 patients (59.5%) were alive and disease-free, and eight patients (19.0%) were alive with recurrent disease or metastasis. Two patients (4.8%) succumbed to the disease 24 months after treatment, one patient (2.4%) was lost to follow-up four years after tonsillectomy, one patient (2.4%) was lost to follow-up after initial chemotherapy and follow-up data were not recorded for five patients (11.9%). The three-, five- and eight-year overall survival rates for the entire group were 86.5, 77.8 and 77.8%, respectively and the three-, five- and eight-year disease-free survival rates were 88.9, 76.2 and 57.2%, respectively. The three-, five-, and eight-year overall survival and disease-free survival rates were similar, however, previous studies have contradicted these results. Li *et al* (10) reviewed 106 cases of extranodal FDCSs located throughout the body and reported that the two- and five-year overall survival rates were 82 and 79%, respectively, and that the two- and five-year disease-free survival rates were 57 and 32%, respectively. Duan *et al* (9) reviewed patients with extranodal FDCSs in the pharyngeal region and found that the recurrence, metastasis and mortality rates were 23.1% (9/39), 20.5% (8/39) and 2.6% (1/39), respectively. The three- and eight-year recurrence-free survival rates of the entire group were 74.8 and 24.6%, respectively (9). As early as 1997, Chan *et al* (24) analyzed the clinicopathological features of 17 cases of extranodal FDCS and found that the overall recurrence, metastasis and mortality rates were 43, 24 and 17%, respectively. A possible reason for differences between the findings of the present study and that of previous studies may be that the present study focused on a single tumor site (the tonsil), thus excluding tumors in other extranodal sites, including the head and neck, pharyngeal region and other sites. Although prognostic factors remain unclear, the FDCS site is important, as patients with FDCSs in the parapharyngeal space exhibit poorer outcomes than those with other affected areas (2). Similarly, intra-abdominal lesions are associated with higher recurrence rates, which impacts patient survival (10).

**Figure 4:**
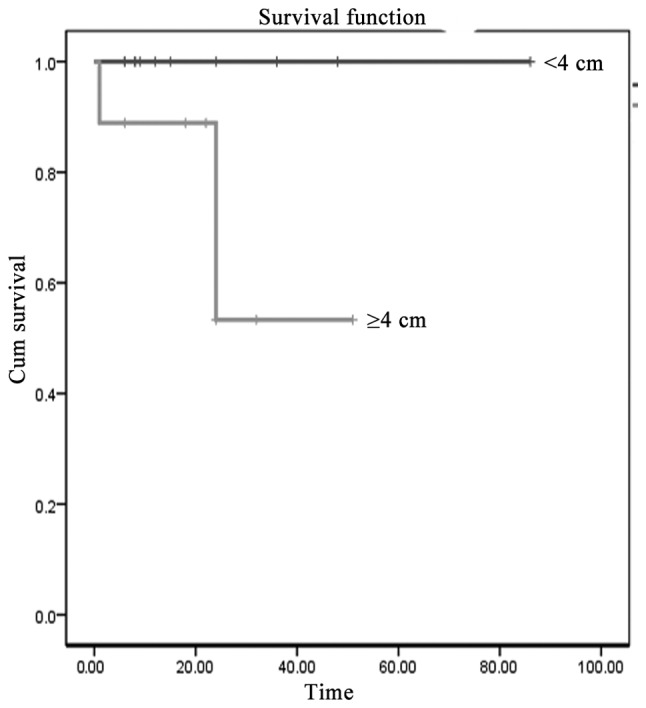
Univariate analysis results revealing that a tumor diameter of ≥4 cm was prognostic (χ^2^=4.634; P=0.031).

Upon univariate analysis, it was found that a tumor diameter of ≥4 cm was prognostic (χ^2^=4.634; P=0.031; Fig. 4; incomplete data was excluded). The five-year survival rate in the combined treatment group (87.9%) was higher than that in the surgery-alone group (62.5%), however, this difference was not statistically significant (P=0.543). No statistically significant differences were identified between survival, recurrence and metastasis. Multivariate analysis revealed no statistically significant differences between survival and tumor size, treatment modality, recurrence or metastasis. In a study of extranodal FDCSs, Hu *et al* (2) also found that patients with large tumors (≥4 cm in diameter) in the pharyngeal region exhibited a worse prognosis compared with those with smaller tumors (2). The study also found that postoperative adjuvant therapy appeared to prolong the disease-free interval compared with surgery alone (2). Leipsic *et al* (39) found that an intra-abdominal tumor location, a tumor diameter of ≥6 cm, a mitotic count of ≥5/10 high-power fields, extensive coagulative necrosis, significant nuclear pleomorphism and a lack of adjuvant therapy, were all significant negative prognostic factors in patients with FDCSs of the mediastinum. However, the present review may be incomplete, due to certain data being unavailable (as can be observed in Table I), which would affect the results.

Tonsillar FDCS is rare and is associated with high rates of recurrence and metastasis. However, the present patient received tonsillectomy and postoperative radiotherapy, and remains alive without disease recurrence or metastasis after 44 months. Therefore, we hypothesize that adjuvant treatment should be prescribed.

## Figures and Tables

**Table I:** Cases of follicular dendritic cell sarcoma of the tonsil in the English literature.

First author, year (ref.)	Age, years/gender	Site	Symptoms and duration	Tumor size	Initial diagnosis	Treatment	Recurrence	Metastasis	Follow-up
Present case	59/M	Right tonsil	Globus sensation	4.6×2.5×2.5 cm	Lymphoma	Tonsillectomy+ postoperative radiotherapy	No	No	NED 32 months after treatment
Hu *et al*, 2013 (2)	36/F	Left tonsil	Oropharyngeal mass slight dysphagia, 1 month	3.0×2.5×1.5 cm	Non specific inflammation	Surgery	Yes. 6 months. Salvage therapy, 4 courses CHOP+ 56 Gy radiotherapy	No	AWD 15 months after initial surgery
	59/F	Left tonsil	Oropharyngeal mass, dysphagia, dyspnea, 2 months	4.5×4×2 cm	Benign tumor	Surgery	17 months	No	DOD 24 months after initial surgery
Kara *et al*, 2013 (5)	72/M	Right tonsil	Painless mass located in the right tonsillar region, discomfort during swallowing food, 2 months. 1-month history of respiratory distress	5×3 cm	NA	Tonsillectomy+ postoperative chemotherapy	NA	NA	24 months. Died after first dose chemotherapy
Mondal *et al*, 2012 (6)	27/M	Left tonsil	Difficulty in swallowing, 3 months	2.8×2.6×2.3 cm	Tonsillar carcinoma or lymphoma	Tonsillectomy+ postoperative radiotherapy	No	No	NED 6 months after treatment
Eun *et al*, 2010 (7)	65/M	Right tonsil	Discomfort during swallowing, 1 week	1×1 cm	NA	Tonsillectomy+ postoperative radiotherapy	No	No	NED 2 years after treatment
Suhail *et al*, 2010 (8)	52/F	Right tonsil	Swelling in the throat and dysphagia, a few weeks	2.5×2 cm	NA	Tonsillectomy+ postoperative chemotherapy	No	No	NED 12 months after treatment
Duan *et al*, 2010 (9)	41/M	Left tonsil	Hypertrophy of the left tonsil, 1 month	3×3×2 cm	NA	Surgery	No	No	NED 9 months after treatment
Li *et al*, 2010 (10)	60/M	Tonsil	NA	5 cm	NA	Surgery+ postoperative radiotherapy	No	No	NED 86 months after treatment
Vaideeswar *et al*, 2009 (11)	50/M	Left tonsil	Dysphagia, 2 months	2×2 cm	NA	Tonsillectomy	No	No	NED 4 years after surgery
McDuffie*et al*, 2007 (12)	59/F	Right tonsil	A mass in the right tonsil and a history of OSAS	4 cm	NA	Surgery+ postoperative radiotherapy	No	No	NED 18 months after treatment
Fan 2007 *et al*, (13)	48/F	Right tonsil	Right tonsil swelling and weight loss	NA	Malignant lymphoma	Tonsillectomy+ postoperative combination chemotherapy+ local radiotherapy	Yes. 15 years. Second treatment, aggressive chemotherapy	Yes	AWD
Aydin *et al*, 2006 (14)	76/F	Left tonsil	A mass in the left tonsil with no symptoms	3.5×3.5×1.5 cm	Tonsillar lymphoma	Tonsillectomy+ postoperative radiotherapy	No	No	NED 4 years after surgery
Clement *et al*, 2006 (15)	27/F	Right tonsil	Dysphagia	4×3×2 cm	Primitive nerve sheath tumor	Tonsillectomy+ selective neck dissection+ postoperative	No	No	NED 6 months after treatment
Shia *et al*, 2006 (16)	69/F	Tonsil	NA	NA	Squamous cell carcinoma	Tonsillectomy+radical neck disection+ postoperative radiotherapy	No	Lung and hilar lymph node metastasis 8 years after surgery	AWD 9 years after treatment treatment
Bothra *et al*, 2005 (17)	40/M	Left tonsil	NA	NA	Carcinoma	Tonsillectomy	No	No	NED 1 year after surgery
	45/M	Right tonsil	NA	NA	Carcinoma	Tonsillectomy	No	No	NED 1 year after surgery
	34/M	Right tonsil	NA	NA	NA	Tonsillectomy	Yes	No	AWD 10 years after surgery
Domínguez-Malagón *et al*, 2004 (18)	48/M	Left tonsil	Dysphagia	1.5×1.5 cm	NA	Tonsillectomy+neck dissection+postoperative radiotherapy	No	No	NED 36 months after treatment
Idrees *et al*, 2004 (19)	70/F	Tonsil	A tonsil mass	NA	Squamous cell carcinoma	Preoperative radiotherapy+ palatopharyngeal tonsil resection+ radical neck dissection	Yes	Yes	Lung and hilar lymph node metastasis 8 years after surgery
Grogg *et al*, 2004 (20)	57/F	Tonsil	NA	NA	NA	None	No	NA	AWD 8 months
Tisch *et al*, 2003 (21)	51/M	Left tonsil	Globus sensation	NA	NA	Tonsillectomy+ postoperative radiotherapy	No	No	NED 5 years after treatment
Biddle *et al*, 2002 (22)	48/M	Right tonsil	Pain in the tonsillar area	3.5×2×2 cm	Chronic tonsillitis	Tonsillectomy	No	No	NED 8 months after surgery
	48/F	Left tonsil	An enlarged, hard, fixed lymph node in the left submandibular area	3.5×3.5×2 cm	Metastatic carcinoma or lymphoma	Tonsillectomy+ radical neck dissection	No	No	NED 6 months after surgery
Vargas *et al*, 2002 (23)	54/F	Left tonsil	A left neck mass and a recent 10 lb weight loss	3 cm	Malignancy	Tonsillectomy+ modified radical neck dissection	No	No	NED 8 months after surgery
Chan *et al*, 1997 (24)	32/M	Right tonsil	Enlarged right tonsil	Tonsil weighing 8 g	NA	Tonsillectomy+ postoperative radiotherapy	Yes. 4.5 years after surgery	Cervical lymph node metastasis 4.5 years after surgery	AWD 4.5 years
Nayler *et al*, 1996 (25)	18/F	Tonsil	Enlarged bilateral tonsil	4×2×2 cm	NA	Bilateral tonsillectomy followed by CHOP chemotherapy	NA	NA	Lost to follow-up after initiation chemotherapy
Perez-Ordoñez *et al*, 1996 (26)	62/F	Tonsil	NA	NA	NA	Surgery	No	No	NED 1 year after surgery
Chan *et al*, 1994 (27)	44/F	Left tonsil	NA	1.5 cm	NA	Surgery	No	No	NED 36 months after surgery

NA, not available; NED, no evidence of disease; AWD, alive with disease; DOD, died of disease; M, male; F, female; OSAS, obstructive sleep apnea with snoring.

**Table II:** Cases of follicular dendritic cell sarcoma of the tonsils in the Chinese literature.

First author, year (ref.)	Age, years/gender	Site	Symptom	Tumor size	Initial diagnosis	Treatment	Recurrence	Metastasis	Follow-up
Shi *et al*, 2004 (28)	37/M	Right tonsil	Globus sensation	1.5×1.5×1 cm	NA	Tonsillectomy+ postoperative chemotherapy	No	No	NED 36 months
Zhang *et al*, 2008 (29)	36/M	Right tonsil	A mass in the right tonsil	NA	NA	Tonsillectomy	No	No	4 years then lost
Chen *et al*, 2009 (30)	NA	Tonsil	Painless mass	NA	NA	Surgery	No	NA	12 months. Alive
Chen *et al*, 2009 (31)	21/F	Right tonsil	Enlarged right tonsil	2.5×2×1.2 cm for 3 years	Spindle cell	Tonsillectomy tumor	NA	NA	NA
Ma *et al*, 2010 (32)	19/F	Tonsil	A mass in the tonsil	1.0×0.6×0.3 cm	NA	Tonsillectomy	NA	NA	NA
	60/M	Right tonsil	A mass in the right tonsil	1.0×0.7×0.7 cm	NA	Tonsillectomy	NA	NA	NA
	40/F	Left tonsil	A mass in the left tonsil	0.8×0.4×0.2 cm	NA	Tonsillectomy	NA	NA	NA
Yin *et al*, 2010 (33)	35/M	Right tonsil	Globus sensation	5.0×3.0×2.5 cm	NA	Tonsillectomy	No	Yes. Lymph node metastasis 1 year after right tonsillectomy	Bilateral neck dissection+ postoperative radiotherapy, NED 39 months after second treatment
Liu *et al*, 2010 (34)	47/F	Right tonsil	Globus sensation	NA	Lymphoma	Tonsillectomy	No	No	NED 10 months after tonsillectomy
Yang *et al*, 2011 (35)	49/F	Right tonsil	Pain in the right tonsil, fever	5.0×4×6 cm	Tonsillitis	Tonsillectomy+ postoperative chemotherapy	No	No	NED 22 months after treatment
Wang *et al*, 2011 (36)	80/M	Right tonsil	Globus sensation	4.6×3×2.8 cm	Tonsillar tumor	Tonsillectomy	No	No	NED 2 years after tonsillectomy
Zhang *et al*, 2012 (37)	43/F	Right tonsil	Globus sensation	3.0×1.5×1.0 cm	NA	Tonsillectomy	NA	NA	NA
Wu *et al*, 2012 (38)	55/F	Left tonsil	Pharyngeal discomfort	NA	Lymphoma	Tonsillectomy	No	No	NED 4 months after tonsillectomy

NED, no evidence of disease; NA, not available; M, male; F, female; OSAS, obstructive sleep apnea with snoring.
